# Moving the Weber Fraction: The Perceptual Precision for Moment of Inertia Increases with Exploration Force

**DOI:** 10.1371/journal.pone.0042941

**Published:** 2012-09-17

**Authors:** Nienke B. Debats, Idsart Kingma, Peter J. Beek, Jeroen B. J. Smeets

**Affiliations:** Research Institute Move, Faculty of Human Movement Sciences, VU University Amsterdam, The Netherlands; University of Muenster, Germany

## Abstract

How does the magnitude of the exploration force influence the precision of haptic perceptual estimates? To address this question, we examined the perceptual precision for moment of inertia (i.e., an object's “angular mass”) under different force conditions, using the Weber fraction to quantify perceptual precision. Participants rotated a rod around a fixed axis and judged its moment of inertia in a two-alternative forced-choice task. We instructed different levels of exploration force, thereby manipulating the magnitude of both the exploration force and the angular acceleration. These are the two signals that are needed by the nervous system to estimate moment of inertia. Importantly, one can assume that the absolute noise on both signals increases with an increase in the signals' magnitudes, while the relative noise (i.e., noise/signal) decreases with an increase in signal magnitude. We examined how the perceptual precision for moment of inertia was affected by this neural noise. In a first experiment we found that a low exploration force caused a higher Weber fraction (22%) than a high exploration force (13%), which suggested that the perceptual precision was constrained by the relative noise. This hypothesis was supported by the result of a second experiment, in which we found that the relationship between exploration force and Weber fraction had a similar shape as the theoretical relationship between signal magnitude and relative noise. The present study thus demonstrated that the amount of force used to explore an object can profoundly influence the precision by which its properties are perceived.

## Introduction

A prominent feature of human behavior is the ability to use tools to perform daily-life tasks, like eating with cutlery or chopsticks. We can perform such tasks even without visual guidance, which highlights the importance of the haptic sense. One can ‘simply feel’ object properties for which we have no dedicated cutaneous sensors. For example, we can perceive the hardness of a ball, the size of a large handheld object, or the viscosity of the liquid in our glass (e.g., [1], [2], [3]). Such haptic perceptual estimates require a force-movement-interaction between the perceiver's hand(s) and the object: when exploring the object the perceiver applies forces to the object, which generally leads to reaction forces that act on the perceiver's hand(s). These forces will result in movement and/or deformation of the object as well as movement and/or deformation of the perceiver's body and skin. As the perceiver increases the magnitude of the exploration forces, the magnitude of the reaction forces will also increase. This entails stronger kinesthetic and tactile afferent signals. In consequence one may wonder whether the magnitude of the exploration force affects the precision of haptic perceptual estimates. This question was addressed in the present study.

To address our question, we studied the perception of moment of inertia. Moment of inertia is an object's ‘angular mass’: its resistance against angular acceleration, just like mass is the resistance against linear acceleration. This object property has been shown to contribute to the perception of geometrical properties of handheld objects (e.g., length) that are rotated like one does with a fly swatter, a tennis racquet, or a hammer (e.g., [4], [5]). Every time one interacts with an object, the object's moment of inertia determines the rotational movement that follows from the forces that are applied to the object. Hence, an object's moment of inertia contributes to its “feel” when manipulated by hand. Participants in the present study rotated a rod, which was mounted on an axis in its center of mass, and judged its moment of inertia. The magnitude of the moment of inertia was adjusted from trial to trial. This perceptual task is equivalent to judging the mass of an object in a zero-gravity environment or in absence of gravitation cues (e.g., [6], [7], [8]).

The relationship between exploration force *F*, moment of inertia *I*, and angular acceleration α provides some basic insights into the sensory information needed to perceive moment of inertia. In the present task, with the rotation axis fixed through the center of mass of the rod, this relationship is as follows:
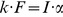
(1)The *k* is a constant that represents the moment-arm of the force. This equation illustrates two things: First, an increase (or decrease) in the exploration force entails a corresponding increase (or decrease) in the magnitude of the rod's angular acceleration. Second, in order to estimate moment of inertia the nervous system requires information about both forces and angular accelerations. Crucially, this information is encoded in neural signals that are subjected to noise [9]. The magnitude of the noise can be assumed to scale linearly with the magnitude of the signal, with a certain offset that indicates an additional constant noise factor. This holds both for efferent signals [10], [11], [12], [13] and afferent signals (i.e., Weber's law; e.g., [14]). Thus, the magnitude of the exploration force determines the magnitude of both the noise in the force information and the noise in the angular acceleration information. This noise necessarily limits the resolution by which the nervous system can estimate moment of inertia, that is, the perceptual precision.

A commonly used measure for perceptual precision is the Weber fraction (e.g., [14]). This measure denotes the percentage difference in stimulus strength that is just noticeable (i.e., correct discrimination in a certain percentage of the trials) to a perceiver. For example, the Weber fraction for mass is about 10% (e.g., [7]), which indicates that an average perceiver can discriminate 1.1 from 1.0 kg, and 2.2 from 2.0 kg. The perceptual estimate of moment of inertia as obtained from noisy sensory information can be regarded as a sample from a Gaussian distribution. The distribution's standard deviation indicates the perceptual precision, with a large value indicating a poor precision and vice versa. Two rods with a slightly different moment of inertia are represented by two Gaussian distributions with a slightly different mean. The distributions' precisions determine their degree of overlap and thus the percentage of trials in which the stimuli are correctly discriminated, as quantified by the Weber fraction. The present study entails such a discrimination paradigm.

**Figure 1:**
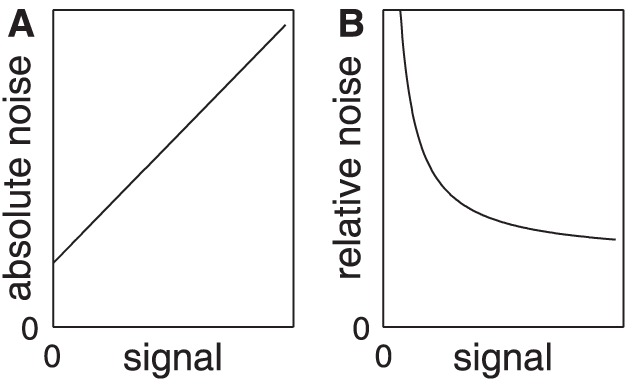
Neural noise. **A:** The absolute noise in a neural signal increases linearly with the magnitude of the signal (signal-dependent noise) in addition to a constant noise factor (signal-independent noise). **B:** The relative noise in a neural signal (defined as the absolute noise divided by the magnitude of the signal) decreases with the magnitude of the signal.

The present study examines how the magnitude of the exploration force influences the precision by which moment of inertia is perceived. This question boils down to: how does neural noise in force information and angular acceleration information affect the perceptual precision for moment of inertia? As it is unknown how the nervous system combines force and angular acceleration information, it is also unknown how the noise in these signals propagates into the perceptual estimate for moment of inertia. The first possibility is that the perceptual precision is determined by the absolute noise, the magnitude of which increases with the magnitude of the exploration force (see Figure 1A). In motor control, it is the absolute noise that determines the precision by which the task can be achieved – an increase in absolute noise causing a decrease in motor precision (e.g., [15]). Similarly, an increase in the absolute noise may cause a decrease in the perceptual precision and thus an increase in the Weber fraction (i.e., Weber fraction ∝ absolute noise). The second possibility is that the perceptual precision is determined by the relative noise, which is the magnitude of the absolute noise divided by exploration force (i.e., a coefficient of variation). An increase in the exploration force leads to a decrease in the relative noise (see Figure 1B) due to the offset in the absolute noise. A decrease in the relative noise may cause an increase in the perceptual precision and thus a decrease in the Weber fraction (i.e., Weber fraction ∝ relative noise). Note that these are plausible yet not exclusive possibilities. The effect of increasing the exploration force may thus range from a linear increase in the Weber fraction (absolute noise) to a gradual decrease in the Weber fraction (relative noise).

To summarize, the present study examines how the magnitude of the exploration force relates to the perceptual precision for moment of inertia. In a series of two psychophysical experiments we manipulated the magnitude of the exploration force and measured the magnitude of the Weber fraction. We first used two magnitudes of exploration force to examine the direction of the relationship, that is, whether the Weber fraction increases or decreases with increasing exploration force. In the second experiment we studied the shape of the relationship in more detail using four magnitudes of exploration force while strictly controlling for potential confounders.

## Methods Experiment 1

In the first experiment, we examined whether the magnitude of the exploration force affects the Weber fraction for moment of inertia. Two levels of force were used in this experiment: low versus high.

### Participants

After being informed about the experimental task, four men and four women (age range: 23–33 years) participated voluntarily in Experiment 1. All participants were naïve about the rationale behind the experiment. The experiment was part of a research program that was approved by the local ethics committee of the Faculty of Human Movement Sciences of VU University Amsterdam. All participants gave written informed consent.

### Apparatus and setup

**Figure 2:**
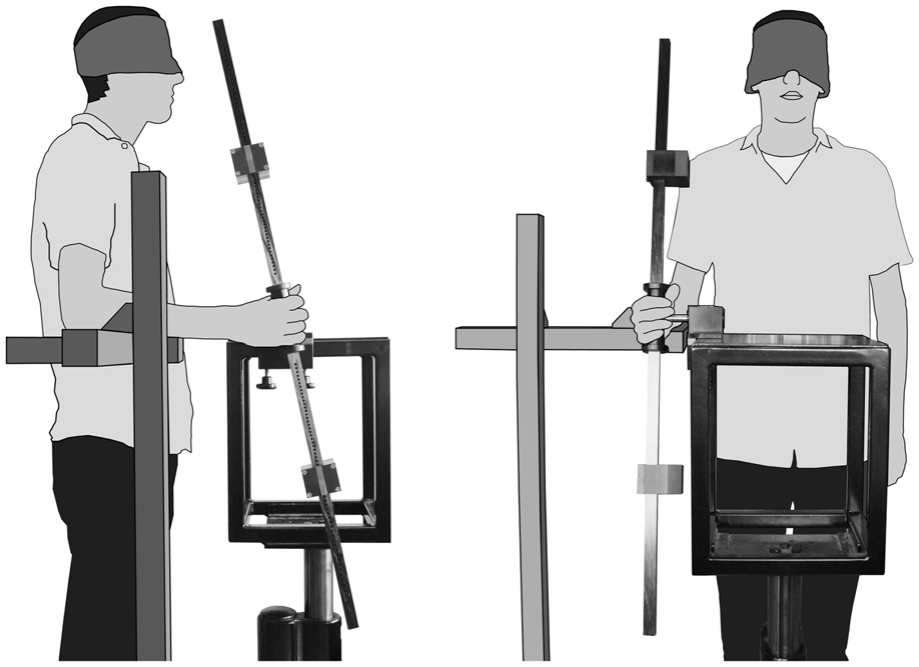
Experimental setup. The setup is illustrated from a side view (left) and a frontal view (right). The rod had one degree of freedom, which allowed rotation to and away from the body. These rotations were made by abduction and adduction of the wrist. Not shown in this figure are the Optotrak cameras and the two markers that were used to record the rod's movements.

A dedicated apparatus was designed and constructed for the experiments (see Figure 2). In essence, the apparatus is a rod that can be rotated around an axis through its center and to which two equal weights are attached whose position along the shaft of the rod can be varied. Fixing the weights close to the axis of rotation results in a relatively small moment of inertia; fixing the weight close to the rod's endpoint results in a relatively large moment of inertia. The weights were always positioned symmetrically relative to the rotation axis so that the rod was balanced in any orientation. The shaft of the rod was a hollow carbon fiber beam (115×2×2 cm), with small holes along its length that served as fixation points for the weights. The weights were rectangular cuboids (5.0×6.0×7.5 cm) with a mass of 0.3064 kg. The positions of the holes were defined such that fixating the weights in subsequent holes always entailed a 3.5% change in moment of inertia. The weights had a lever with a spring on one side and a pellet on the other; the pellet neatly fitted in the fixation hole, thus fixating the weight on the shaft. The spring ensured a fast release of the pellet so that it took only a few seconds to change the position of the weights and thus the rod's moment of inertia. The rotation axis in the center of the rod was attached to a solid base using double bearing for minimal friction. Around the center of the rod there was a synthetic cylindrical handle (10 cm in length and 3 cm in diameter) for a convenient grip.

The participants were either standing or seated – whichever they preferred – with their right upper-arm vertical along their body and their right forearm on a horizontal armrest. The height of the armrest was customized to the individual participants. The rod was mounted on a heavy pole that was fixed firmly to the ground. The pole's height could be adjusted such that the rod's center could be set at a comfortable height (about equally high as the center of the wrist) for each individual participant. Participants were blindfolded throughout the experiment.

An Optotrak 3020 camera system was used to record the position of two infrared markers on the rod with a sample frequency of 200 Hz. The recording of these time series was manually started and stopped by the experimenter. The recordings were stored automatically on a computer for offline analysis.

### Design

The Weber fractions for moment of inertia were determined with a two-alternative forced choice paradigm, using the method of constant stimuli. Participants' task was to sequentially explore two stimuli (i.e., the rod with two magnitudes of moment of inertia) and reported which stimulus had a higher moment of inertia. In between the two periods of exploration there was a brief period (∼3 seconds) in which the experimenter changed the stimulus by adjusting the position of the weights. One of the two stimuli was the reference stimulus (68.66·10^−3^ kg.m^2^, unknown to the participants). The other was the test stimulus, which had one out of twelve possible magnitudes (55.85, 57.81, 59.83, 61.93, 64.09, 66.34, 71.06, 73.55, 76.12, 78.79, 81.55, 84.40 kg.m^2^·10^−3^). This range of stimuli (±23% around the reference) was chosen based on pilot measurements with four participants. To determine one Weber fraction, each combination of reference and test stimulus was presented ten times, yielding 120 trials in total. The order of the test stimuli was randomized; for each stimulus the reference was presented first in half of the trials. The 120 trials were measured in one session, which took about 2 hours to complete. Short breaks were generally made after 40 and 80 trials. Throughout the session a radio was turned on (playing at low volume) to keep the participants energetic and motivated.

All participants performed two sessions of the experiment: once with the instruction to use a low level of force to rotate the rod (the *low* force condition) and once with the instruction to use quite-a-bit-of force while staying below the level of force that would induce muscular fatigue (the *high* force condition). Prior to the experiment participants practiced rotating the rod for a few minutes to select their individual reference for low force and high force. Their only restriction was to firmly hold the rod at its handle without squeezing it. Regarding the experimental trials, participants were instructed to apply a force that approximated their reference force level. They were explicitly instructed to focus on the discrimination task and not on reproducing the exact reference force level. The two force conditions were performed on separate days; their order was counterbalanced over the participants.

### Procedure

First, the height of the experimental setup was customized for the participants. Second, participants were acquainted with the task – discriminating the object's moment of inertia – as follows: the experimenter explained that moment of inertia is the resistance of an object against angular acceleration and that this object property is similar to what mass is for linear acceleration. Next, participants rotated the rod twice, once with a low example moment of inertia (22·10^−3^ kg.m^2^) and once with a high example moment of inertia (186·10^−3^ kg.m^2^). They were asked to feel “which rod felt heavier to rotate”. After this illustration, all participants reported that they understood what an object's moment of inertia is. Third, participants were instructed on the level of force they were to use. Last, participants signed the informed consent form, they were blindfolded, and the first trial commenced.

### Analysis

**Figure 3:**
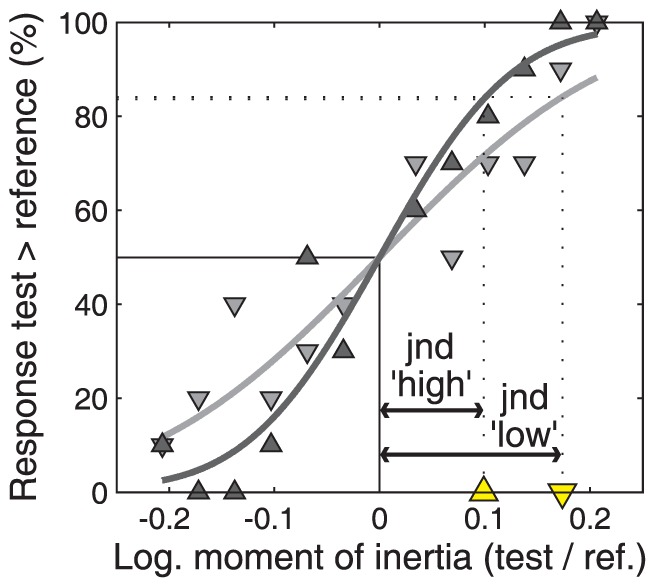
Psychometric function. An exemplary participant's responses in the low and high force conditions and the psychometric curves (cumulative Gaussians) fitted to the responses. On the *x*-axis are the log-transformed values for moment of inertial for the twelve test stimuli relative to the reference stimulus. On the *y*-axis are the participant's responses, that is, the percentage of trials in which each test stimulus was judged to have a larger moment of inertia than the reference stimulus. The light gray downward pointing triangles and dark gray upward pointing triangles correspond to the responses in the low and high force condition, respectively. The curves' standard deviations (jnd) are indicated with the yellow triangles on the *x*-axis. The Weber fraction is defined as the jnd divided by the reference stimulus' moment of inertia. See section “analysis” for further details.

Participants reported which of the two stimuli that they explored per trial had a higher moment of inertia. We fitted psychometric functions (see Figure 3) to these data using psignifit version 2.5.6 (see http://bootstrap-software.org/psignifit/), a software package which implements the maximum-likelihood method described by Wichmann and Hill [16]. On the *x*-axis of the psychometric curve was the natural logarithm of test stimulus's moment of inertia divided by the reference stimulus's moment of inertia. On the *y*-axis was the percentage of trials in which the moment of inertia was judged to be larger in the test stimulus than in the reference stimulus.

The psychometric functions were defined as cumulative Gaussian distributions because there was no reason to assume that the data were other than normally distributed. We first fitted the psychometric functions with three free parameters: 1) the psychometric functions' point of subjective equality (pse; the mean of the Gaussian distribution), 2) the psychometric functions' just noticeable difference (jnd; the standard deviation of the Gaussian distribution), and 3) the deviation of the psychometric functions' lower and upper bounds from 0 and 1, respectively. This parameter was free to vary between 0–0.05 in order to account for occasional lapses [16]. This first fit revealed no unexpected biases – the pse did not differ significantly from zero in both force conditions (*t*-tests, *p*-values >.10). Hence, we fixed the pse to zero and fitted the psychometric functions with two free parameters. The Weber fractions were determined as the jnd divided by the reference stimulus's moment of inertia. High Weber fractions indicate poor precision and vice versa.

Participants were free to select their own reference for low and high force conditions. The Optotrak kinematic recordings were analyzed to determine *angular acceleration* and *exploration force*. We first calculated the rod angle time series, which describes the angle from the horizontal plane to the vector between the two markers on the rod. The mean absolute *angular acceleration* of the rod was determined from the second time derivative of the rod angle. *Exploration force* was calculated with equation 1, using half the width of each participant's hand as the moment-arm ‘*k*’. Hand-width was measured at the metacarpophalangeal joints of the four fingers (i.e., excluding the thumb). This single force measure reflects all forces and force couples that the perceiver applies to the rod. In addition we determined the peaks in the rod angle time series to determine the number of *movement cycles* and the *movement time* for each stimulus. All measures were first determined for the two subsequent stimuli in a trial separately; subsequently they were averaged to obtain one value per measure per trial.

### Exclusion criterion and statistical analysis

In order to accurately estimate the psychometric functions and the corresponding Weber fractions, the range of presented stimuli (i.e., ±23%) should approximate the magnitude of the Weber fraction. If the estimated Weber fraction exceeded this range by a factor two (i.e., >46%), we rejected it as an unreliable estimate. Based on this criterion we had to exclude one participant in experiment 1 for whom the estimated Weber fraction was 75% in the low force condition.

All statistical analyses were performed with paired-samples *t*-tests. First, we examined whether participants had successfully followed the instructions to produce two different levels of exploration force. We also verified that these force levels corresponded to two different magnitudes of the angular acceleration. Second, we examined whether the level of exploration force influenced the perceptual precision by analyzing the difference in Weber fraction between the force conditions. Last, we examined whether the force conditions differed in the number of movement cycles and in movement time.

## Results Experiment 1

### Exploration

**Figure 4:**
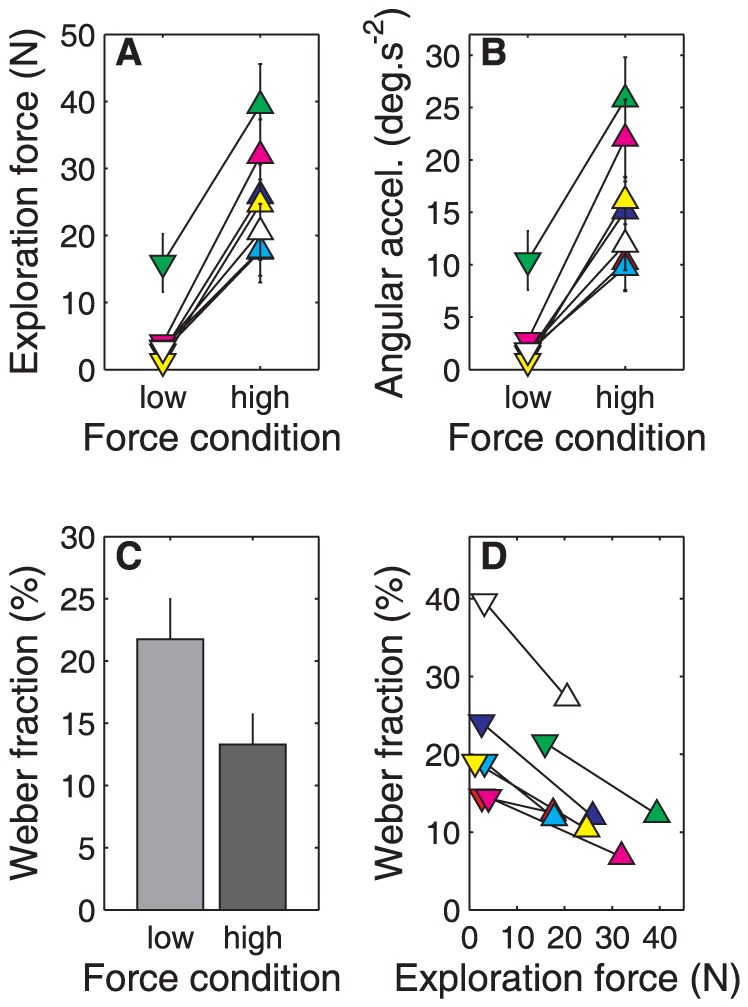
Results of Experiment 1. **A:** Magnitude of the exploration force for each individual participant in both force conditions. The colors indicate individual participants; the error bars indicate one standard deviation. The yellow symbols indicate the exemplary participant in Figure 3. **B:** Magnitude of the angular acceleration (details as in A). The graphs in A and B are very similar because force and angular acceleration are linearly related (see equation 1). Yet, there are small differences between panels A and B because, for example, a high exploration force in a stimulus with a low moment of inertia leads to a higher angular acceleration than if it were a stimulus with a high moment of inertia. **C:** Average Weber fractions in the low force and high force conditions. The error bars indicate the standard error over participants. **D:** Relationship between exploration force and Weber fraction for each individual participant (color coding as in A and B).

Figure 4A shows that participants adequately followed the instruction to use two different force levels. Paired-samples *t*-tests revealed a significantly lower exploration force in the low force condition (4.7 N) than in the high force condition (25.4 N) (*t*(6) = −10.86, *p*<.001). In congruence, the angular acceleration was significantly lower in the low force (2.9°/s^2^) than in the high force condition (15.9°/s^2^) (*t*(6) = −8.32, *p*<.001; see Figure 4B).

### Perceptual precision

Figure 4C shows the average Weber fractions in the two conditions in which participants used either a low or a high level of force to rotate the rod. A paired-samples *t*-test revealed that the Weber fractions were significantly higher in the low force condition (22%) than in the high force condition (13%) (*t*(6) = 6.44, *p* = .001). This result indicates that participants were poorer in discriminating moments of inertia in the low force condition than in the high force condition. Thus, exploration with a large magnitude of the exploration force resulted in better perceptual precision for moment of inertia than exploration with a small magnitude of the exploration force. Figure 4D illustrates the relationship between exploration force and the Weber fraction; for each individual participant there was a decrease in the Weber fraction with an increase in exploration force.

### Potential confounders

Participants were constrained in the amount of force they used to rotate the rod, but not in the amount of time and the number of completed movement cycles. Yet, more exploration time and a larger number of movement cycles may have improved the perceptual performance, thus confounding the Weber fractions. Paired-samples *t*-tests revealed that participants had a smaller number of movement cycles and a longer movement time (3.9 cycles and 5.3 seconds) in the low force condition than in the high force condition (5.6 cycles and 3.3 seconds) (*t*(6) = −3.7, *p* = .01 and *t*(6) = 7.47, *p*<.001). Thus, the perceptual performance in the high force condition might have benefitted from more movement cycles, whereas performance in the low force condition might have benefitted from a longer movement time.

## Discussion Experiment 1

In Experiment 1 we examined whether the perceptual precision for moment of inertia increased or decreased with in an increase in the magnitude of the exploration force. Participants were well able to rotate the rod with two different magnitudes of the exploration force. In congruence, the rod was rotated with two different magnitudes of angular acceleration. Thus, we can assume that both the force information and the angular acceleration information contained more noise in the condition with a large magnitude of the exploration force. Despite this larger absolute noise we found that the Weber fraction was lower in this condition, indicating that the perceptual estimates were more precise. This finding suggests that the Weber fraction for moment of inertia is not constrained by the absolute noise. Instead it may be constrained by the relative noise (see Figure 1).

The hypothesis that the Weber fraction is proportional to the relative noise leads to very specific predictions for the shape of the relationship between the exploration force and the Weber fraction. As Figure 1B illustrates, we predict that the relationship is a curve that is characterized by an initial fast decline in the Weber fraction followed by stabilization at a constant value. More specifically, we predict that the curved relationship between the exploration force and the Weber fraction corresponds to a linear relationship between exploration force on the *x*-axis and the product of the Weber fraction and the exploration force on the *y*-axis. Such a transformation is equivalent to transforming Figure 1B into 1A. Hence, we predict a linear relationship between the exploration force and the transformed Weber fraction that has a positive slope and a positive intercept.

There were two potential confounders in the Experiment 1: both the number of movement cycles and the movement time differed between conditions. Participants used a larger number of movement cycles and a shorter movement time in the high force condition. These opposite effects reflect that participants merely increased movement frequency, rather than movement amplitude, when asked to use more force to rotate the rod. One could argue that the Weber fraction is, in principle, not determined by a lack of information but by a limited resolution in that information. Nevertheless, we might have overestimated the Weber fractions in the high force condition if the shorter movement time in this condition was insufficient to employ the full resolution. Similarly, we might have overestimated the Weber fraction in the low force condition if the lower number of movement cycles was insufficient to employ the full resolution. Overestimating the Weber fraction in the low force condition might have caused us to overestimate the influence of exploration force. In order to conservative in Experiment 2, we controlled the number of movement cycles.

To summarize, in Experiment 2, we examined the hypothesis that the Weber fraction for moment of inertia is proportional to the relative noise in the force and angular acceleration information. To exclude a potential overestimation of the effect of exploration force, we controlled the number of movement cycles.

## Methods Experiment 2

In Experiment 2, the Weber fraction for moment of inertia was measured for four different levels of exploration force, referred to as *F1*, *F2*, *F3*, and *F4*. Overall, the methods were identical to Experiment 1 – the exceptions are described here.

### Participants

After being informed about the experimental task, two men and four women (age range: 25–44 years) volunteered for Experiment 2. All participants gave written informed consent. They were naïve about the rationale behind the experiment (none of them had participated in Experiment 1).

### Design

We determined Weber fractions for moment of inertia at four different levels of exploration force. Thus, each participant performed four sessions of the task (see Methods experiment 1). The four non-overlapping force categories were 2.5 to 5 N (*F1*), 5 to 10 N (*F2*), 10 to 20 N (*F3*), and 20 to 35 N (*F4*). Prior to each session there was a dedicated two-minute force-practice trial in which participants were asked to rotate the rod with about 30° amplitude and to synchronize the moment with an auditory metronome. The metronome had a frequency of 30, 45, 65, or 85 beats-per-minute, and the rod's moment of inertia was at the reference magnitude. Participants were instructed to memorize the level of force required to perform the prescribed movement and to maintain approximately this amount of force throughout the experimental session. They were instructed that their performance on the discrimination task was more important than the accuracy by which they produced the required force level. In addition, no metronome pacing was provided in the experimental trials to ensure that participants would focus on the perceptual discrimination task instead of timing accuracy. To prevent drift in the force levels, we repeated a short (∼30 seconds) force-practice trial prior to each block of 10 trials. In addition to prescribing movement frequency, we constrained the number of rotation cycles by means of verbal instruction (i.e., “start” and “stop”). Participants completed six cycles for the first stimulus in a trial; for the second stimulus they were allowed to complete less cycles – but not more – if they were certain about their answer. The four conditions were performed on separate days; their order was counterbalanced over the participants.

### Exclusion criterion and statistical analysis

The main experimental challenge in Experiment 2 was to constrain the level of exploration force to the four categories as we defined them. The auditory metronome largely defined the force levels, yet they were also influenced by individual differences in movement amplitude and movement fluency. Out of the 6 (participants)×4 (force category) = 24 sessions, four participants had no data in one force category and double data in another category. For one participant we approved the data because the achieved force level (10.06 N) only marginally exceeded the intended level *F2* (5 to 10 N). For the other three participants there was a larger deviance. Hence, two participants repeated *F4* and one participant repeated *F3*. For the force categories in which we had double data, we used all data (i.e., 240 instead of 120 trials) to determine the Weber fraction.

One-way ANOVAs were conducted to test whether the exploration force and angular acceleration differed between the four force conditions. This ANOVA was also used to examine whether the Weber fraction for moment of inertia differed between the force conditions. Last, we examined whether the relationship between the exploration force and the Weber fraction had a curve similar to the theoretical curve for relative noise. To this aim we determined a *transformed Weber fraction* as the product of the Weber fraction and the exploration force. We predicted a linear relationship between the exploration force and the transformed Weber fraction that has a positive slope and intercept. We determined this relationship with a linear regression analysis for repeated measures (i.e., generalized estimating equations).

## Results Experiment 2

### Exploration

**Figure 5:**
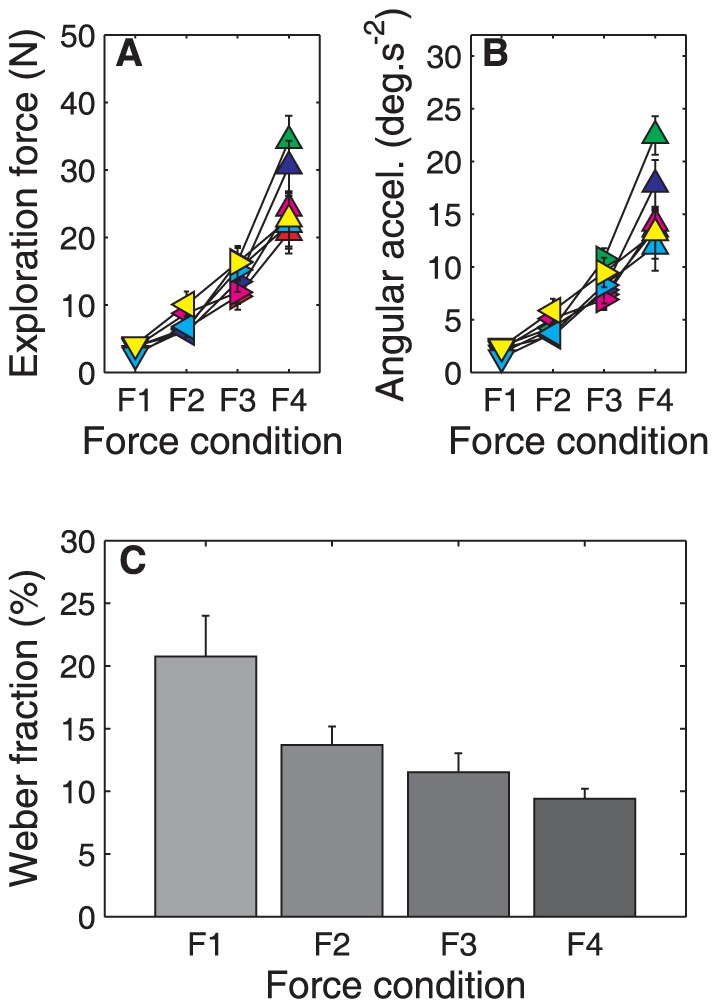
Results of Experiment 2. **A:** Magnitude of the exploration force for each participant in the four force conditions. The colors indicate individual participants; the error bars indicate one standard deviation. **B:** Magnitude of the angular acceleration (details as in A). **C:** Average Weber fraction in the four force conditions. The error bars indicate the standard error over participants.

Figure 5A illustrates that participants adequately followed the instruction to use four different force levels. The ANOVA revealed a significant main effect of force condition on *exploration force* (*F*_3,15_ = 61.15, *p*<.001). Post-hoc pairwise comparisons demonstrated that all four force levels *F1* (3.5 N), *F2* (7.4 N), *F3* (14.1 N), *F4* (25.8 N) were significantly different from each other (*p*-values<.01; see Figure 5A). Similarly, the ANOVA on *angular acceleration* revealed a main effect of force conditions (*F*_3,15_ = 49.46, *p*<.001). Post-hoc pairwise comparisons revealed that the accelerations in all four force levels *F1* (2.1°/s^2^), *F2* (4.4°/s^2^), *F3* (8.4°/s^2^), and *F4* (15.5°/s^2^) were significantly different from each other (*p*-values<.01; see Figure 5B).

### Perceptual precision

The ANOVA on the Weber fractions revealed a main effect of force level (*F*_3,15_ = 10.12, *p* = .001). Post-hoc pairwise comparisons revealed that the Weber fractions were higher in *F1* – the conditions with the lowest force – than in the other three conditions (*p*-values<.05). Furthermore, the Weber fraction was larger in *F2* than in *F4* (*p*<.05). These results are illustrated in Figure 5C.

### Perceptual precision and exploration

**Figure 6:**
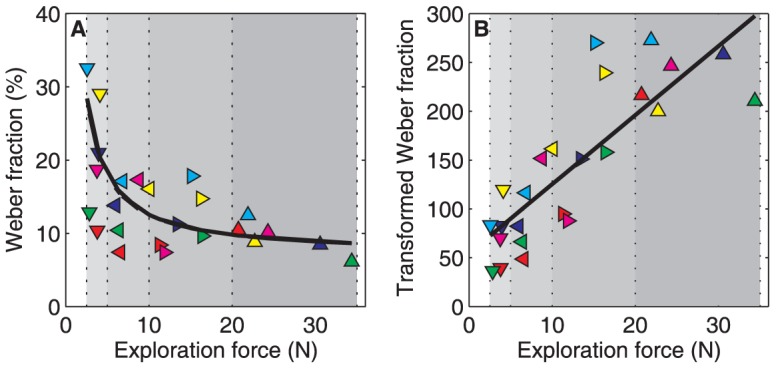
Relationship between force and Weber fraction. **A:** Relationship between the exploration force and the Weber fraction. Colored symbols indicate the individual participants (same color coding as in Figure 5). The grey shaded areas indicate the four force categories *F1*, *F2*, *F3*, and *F4*. The black solid curve corresponds to the best-fit linear regression line shown in panel B. **B:** Relationship between the exploration force and the transformed Weber fraction (i.e., the product of the exploration force and the Weber fraction) (details as in A). The black solid line represents the best-fit linear regression line.

Figure 6A illustrates, for all individual participants, the magnitudes of the exploration forces and the corresponding Weber fractions for moment of inertia. Our hypothesis – the Weber fraction is proportional to the relative noise – predicts a specific decline in the Weber fraction with increasing exploration force (see Figure 1B). More specifically, it predicts that the relationship between the exploration force and the transformed Weber fraction (i.e., the product of Weber fraction and the exploration force) is linear with a positive slope and a positive intercept. This relationship is illustrated in Figure 6B. Linear regression analysis revealed a best fit (black solid line) with a slope (7.07) significantly different from zero (*p*<.001; 95% confidence interval: 5.23–8.91) and an intercept (54.62) significantly different from zero (*p*<.001; 95% confidence interval: 27.59–81.66). The solid black curve in Figure 6A corresponds to the best linear fit in Figure 6B.

The intercept in Figure 6B is responsible for the steepness of the curve in Figure 6A. A low intercept relates to a very steep decline in Weber fraction at low exploration forces, whereas a high intercept relates to a more gradual decline in Weber fraction. Regardless of the rate of decline, the Weber fraction asymptotes to a constant Weber fraction for the higher exploration forces. There is no reason to assume a fixed rate of decline for all participants, and indeed, Figure 6 seems to suggest that there are individual differences. Two participants (red and green symbols) seem to have already reached the asymptote in *F1*; two other participants (dark blue and magenta symbols) reached the asymptote in *F3*; and the last two (light blue and yellow symbols) reached the asymptote only in *F4*.

## General Discussion

The objective of this study was to examine how the magnitude of force applied during object exploration influences the precision of haptic perceptual estimates. To this aim, we determined the perceptual precision – as quantified by the Weber fraction – for moment of inertia under different force conditions. An increase in exploration force coincides with an *in*crease in the absolute noise on the information that is needed to estimate moment of inertia (i.e., exploration force and angular acceleration), but a *de*crease in the relative noise. In Experiment 1 we found that an increase in the exploration force caused a decrease in the Weber fraction, suggesting that the perceptual precision is constrained by the relative noise. Experiment 2 was designed to further examine this hypothesis by having the participants perform the perceptual task four times using four separate force magnitudes. The relationship between the exploration force and the Weber fraction had a similar curve as the theoretical relationship between signal magnitude and relative noise. This finding strengthens the hypothesis that the perceptual precision for moment of inertia is determined by the relative noise in force and angular acceleration information. The present study thus demonstrates that the amount of force used to explore an object can have a profound influence on the precision by which its properties are perceived.

In the first experiment we identified an unequal number of movement cycles between the two force conditions (3.9 vs. 5.6 cycles) as a potential confounder for the magnitudes of the Weber fractions. The lower number of movement cycles in the low force condition could have resulted in a poorer precision and thus in an overestimated Weber fraction. Hence, we constrained to number of movement cycles to six in the second experiment. The effect observed in Experiment 1 – a decrease in the Weber fraction with an increase in the exploration force – was still observed in the second experiment. Moreover, the two conditions with corresponding force levels in Experiment 1 (low force: 4.7±0.6 N; mean force ± SD) and Experiment 2 (*F*1: 3.5±0.6 N) revealed very similar Weber fractions (low force: 21.7±8.6% and *F*1: 20.7±8.7%). These observations suggest that the unequal number of movement cycles in Experiment 1 did most likely not affect the obtained Weber fractions.

Previous reports about the Weber fraction for moment of inertia are scarce and mixed in outcome. The three studies cited here based the Weber fractions on a 75%-correct threshold. To allow for a comparison with our findings, we adjusted these Weber fractions into the values that correspond to an 84%-correct threshold (i.e., one standard deviation). These adjusted Weber fraction are report here. The current Weber fractions were smaller than the 86–168% reported by Ross and Benson [17], and the 45% reported by Kreifeldt and Chuang in their Experiment 1 [18]. The present findings are consistent with the 15% reported by Knowles and Sheridan [19]. The present study differs from these previous studies in that our participants used their full hand to grasp a relatively large object. The previous studies, in contrast, used much smaller stimuli that were grasped between thumb and index finger. Kreifeldt and Chuang [18] used large objects and a full-hand grip in their Experiment 2. Yet it seems that the grip was not in the stimuli's center of mass, so that the stimuli must have differed in both their moment of inertia and their first moment of mass distribution, which may have confounded the discrimination threshold. At the hand-rod interface, the exploration force leads to a pressure that is encoded by the mechanoreceptors (the pressure sensors) in the skin of the hand. The density of the mechanoreceptors is much higher in the fingertips than in the palm of the hand [20]. Therefore, it is surprising that the perceptual precision in our full-hand task was at least as good as in the fingertip task. This may suggest that the role of tactile force detection in the perception of moment of inertia was only minor. Instead, the efferent motor commands may play a major role.

The present study was not designed to examine the mechanisms by which the nervous system obtains a perceptual estimate for moment of inertia. Yet we can speculate on it. Possibly, participants might not have estimated moment of inertia at all. Instead, they might have performed the perceptual task by keeping the force constant for the two consecutive stimuli per trial to judge the difference in resulting angular acceleration, or vice versa. To check for such a strategy we determined for each trial the ratio in the exploration force and the ratio in the angular acceleration for the stimulus with the smaller moment of inertia relative to the stimulus with the larger moment of inertia. The natural logarithm of the force-ratio was significantly smaller than zero in all force conditions (six *t*-tests: *p*<.01); the natural logarithm of the angular acceleration-ratio was significantly larger than zero (six *t*-tests: *p*<.01). This indicates that on average participants used a bit less force and obtained a bit higher angular acceleration for the stimulus with the smaller moment of inertia. Thus, neither force nor angular acceleration was kept constant. Nevertheless, participants may have discriminated force or angular acceleration instead of moment of inertia. If so, trials with a positive force-ratio or a negative angular acceleration-ratio should have had an *in*correct discrimination. This prediction was refuted by the data: the stimuli were correctly discriminated in the majority of the positive force-ratio trials (mean: 80%, between-condition SD: 5%) as well as the negative angular acceleration-ratio trials (mean: 77%, between-condition SD: 4%). Thus, we can conclude that participants truly derived an estimate for the rod's moment of inertia.

A potential mechanism for the nervous system to estimate moment of inertia is the use of an internal forward model [21] that predicts angular acceleration based on an efference copy of the motor commands sent to the arm muscles and an assumed magnitude of the rod's moment of inertia (I_i_). Any discrepancies between the predicted and actual angular accelerations could then be used to derive an updated estimate for moment of inertia (I_i+1_). For example, the updating could be based on the actual (*α*_actual_ with standard deviation σ*_α_*) and predicted angular acceleration (*α*_predicted_) as: I_i+1_ = I_i_ · (*α*_actual_±σ*_α_*)/*α*_predicted_. The precision by which moment of inertia can be updated is thus given by σ*_α_*/*α*_predicted_, which is the relative noise in the angular acceleration. Such a mechanism could thus explain our experimental observations. In contrast, updating that is based on the difference between the actual and predicted movements (i.e., (*α*_actual_±σ*_α_*)−*α*_predicted_) would result in updating with a precision that is given by σ*_α_*, which is the absolute noise in the angular acceleration. Such updating is hence inconsistent with the present data.

The previous paragraph illustrates that it is the exact manner in which force information and angular acceleration information are combined that defines how the noise in these signals propagates into the noise of the perceptual estimate. Our findings seem to suggest a neural mechanism in which the relative noise determines the final perceptual precision. Although this is highly speculative yet, it is possible that such a mechanism might also underlie other haptic perceptual tasks or even perception in general.

The precision of sensory information is of great relevance to the human observer within the theoretical framework of optimal cue integration. If multiple information sources provide cues for the same perceptual estimate, these redundant cues can be integrated by the central nervous system. This integration process can be regarded as a weighted averaging of the separate cues in which the cues' weights are scaled to their precision (e.g., [22], [23]). If the amount of force used to explore an object influences the precision of haptic cues, as the present findings suggest, it will also affect cue weighting. Perceivers could thus exploit force-dependent precision by controlling their exploration style to promote certain cues over others. Such a strategy could lead to a stereotypical coupling between haptic task and exploration style [2], [24], and to specific relationships between movement parameters and cue weighting [25].

We have recently developed a model that explains haptic length perception of hand-held rods as an instance of cue integration [26]. Judgments of rod length depend on the sensory estimate of – at least – three information sources: the rod's mass, its static moment (i.e., its first moment of mass distribution), and its moment of inertia (e.g., [5]). It was found that the weighting of these information sources in the length estimates was different for different movement instruction [26], [27], [28]. These findings stand in sharp contrast with the inertia tensor hypothesis, which assumes that the inertia tensor is the only cue for rod length irrespective of the exploratory movements (e.g., [4]). In our model we determined the length cues' weights from the amount of force that was exerted during exploration. The present findings endorse this method for moment of inertia. For mass perception, reports are inconclusive about the benefit of increased force (i.e., increased linear acceleration or ‘jiggling’) on the perceptual precision [29], [30]. For both mass and static moment it would therefore be interesting to conduct a similar isolated test as the one conducted in the present study.

To conclude, the present study has demonstrated that an increase in the magnitude of the exploration force relates to an increase in the perceptual precision for moment of inertia. This finding suggests that the perceptual precision is determined by the relative noise in the sensory information, rather than by the absolute noise.
